# An easier life to come for mosquito researchers: field-testing across Italy supports VECTRACK system for automatic counting, identification and absolute density estimation of *Aedes albopictus* and *Culex pipiens* adults

**DOI:** 10.1186/s13071-024-06479-z

**Published:** 2024-10-02

**Authors:** Martina Micocci, Mattia Manica, Ilaria Bernardini, Laura Soresinetti, Marianna Varone, Paola Di Lillo, Beniamino Caputo, Piero Poletti, Francesco Severini, Fabrizio Montarsi, Sara Epis, Marco Salvemini, Alessandra della Torre

**Affiliations:** 1 Department of Public Health and Infectious Diseases, Sapienza University of Rome, Rome, Italy; 2 Center for Health Emergencies, Fondazione Bruno Kessler, Trento, Italy; 3 Department of Infectious Diseases, Istituto Superiore Di Sanità, Rome, Italy; 4 Department of Biosciences and Pediatric Clinical Research Center “Romeo Ed Enrica Invernizzi”, University of Milan, Milan, Italy; 5 Department of Biology, University of Naples Federico II, Naples, Italy; 6 Istituto Zooprofilattico Sperimentale Delle Venezie, Legnaro, Italy

**Keywords:** Mosquito trap, Optical sensor, Machine learning, Automatic identification, *Aedes albopictus*, *Culex pipiens*, Genus and sex classification, Mosquito monitoring, Capture Rate

## Abstract

**Background:**

Disease-vector mosquito monitoring is an essential prerequisite to optimize control interventions and evidence-based risk predictions. However, conventional entomological monitoring methods are labor- and time-consuming and do not allow high temporal/spatial resolution. In 2022, a novel system coupling an optical sensor with machine learning technologies (VECTRACK) proved effective in counting and identifying *Aedes albopictus* and *Culex pipiens* adult females and males. Here, we carried out the first extensive field evaluation of the VECTRACK system to assess: (i) whether the catching capacity of a commercial BG-Mosquitaire trap (BGM) for adult mosquito equipped with VECTRACK (BGM + VECT) was affected by the sensor; (ii) the accuracy of the VECTRACK algorithm in correctly classifying the target mosquito species genus and sex; (iii) *Ae. albopictus* capture rate of BGM with or without VECTRACK.

**Methods:**

The same experimental design was implemented in four areas in northern (Bergamo and Padua districts), central (Rome) and southern (Procida Island, Naples) Italy. In each area, three types of traps—one BGM, one BGM + VECT and the combination of four sticky traps (STs)—were rotated each 48 h in three different sites. Each sampling scheme was replicated three times/area. Collected mosquitoes were counted and identified by both the VECTRACK algorithm and operator-mediated morphological examination. The performance of the VECTRACK system was assessed by generalized linear mixed and linear regression models. *Aedes albopictus* capture rates of BGMs were calculated based on the known capture rate of ST.

**Results:**

A total of 3829 mosquitoes (90.2% *Ae. albopictus*) were captured in 18 collection-days/trap/site. BGM and BGM + VECT showed a similar performance in collecting target mosquitoes. Results show high correlation between visual and automatic identification methods (Spearman *Ae. albopictus*: females = 0.97; males = 0.89; *P* < 0.0001) and low count errors. Moreover, the results allowed quantifying the heterogeneous effectiveness associated with different trap types in collecting *Ae. albopictus* and predicting estimates of its absolute density.

**Conclusions:**

Obtained results strongly support the VECTRACK system as a powerful tool for mosquito monitoring and research, and its applicability over a range of ecological conditions, accounting for its high potential for continuous monitoring with minimal human effort.

**Graphical Abstract:**

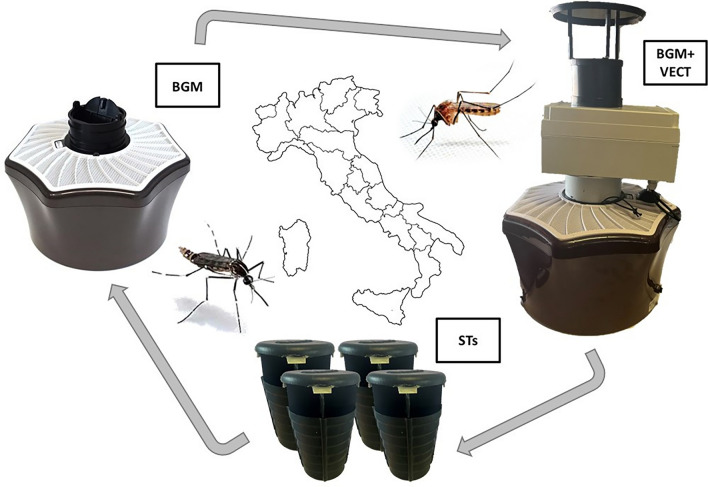

**Supplementary Information:**

The online version contains supplementary material available at 10.1186/s13071-024-06479-z.

## Background

Mosquitoes (Diptera: Culicidae) are vectors of several human pathogens, such as malaria parasites, and dengue (DENV), chikungunya (CHIKV), yellow fever, Zika and West Nile (WNV) viruses, causing diseases resulting in approximately 700,000 deaths/year worldwide [1–3]. Mosquito-borne diseases (MBDs) are becoming a major public health concern also in Europe, where an increasing number of autochthonous cases of arboviruses and a few outbreaks have occurred over the last two decades [4]. *Culex pipiens*, the native vector species, has been responsible for the endemic transmission of WNV in Europe since 2010, particularly in central and southeastern Europe, with the largest outbreak in 2018 [5–7]. In Italy, many WNV endemic cases, some even fatal, are reported each year, mainly in northern Italy [8, 9]. In addition, the risk of exotic arbovirus transmission by the invasive vector species *Aedes albopictus* is rising [10]. This was revealed by two large CHIKV outbreaks occurring in Italy in 2007 and 2017 [11–13] and by several DENV outbreaks in Croatia, France and Spain, including three outbreaks in Italy in 2020 and 2023, with 93 cases in three separate regions [14–18]. For these reasons, effective mosquito control is important to mitigate the impact of MBDs [19, 20].

Effective vector monitoring is an essential prerequisite to acquire data to optimize control strategies and interventions as well as for evidence-based risk predictions [21, 22]. However, conventional entomological monitoring methods are labor- and time-consuming and do not allow high temporal or spatial resolution, as they can hardly be implemented on a large scale and for an entire season [23]. These methods require qualified staff to check traps in the field at regular intervals, collect samples, make entomological identifications and analyze data. The time required for these different steps hinders large-scale routine and standardized data collections as well as the scaling up of timely application of control measures.

The exploitation of specific sensors coupled with machine learning (ML) technologies has the potential to address these major limitations and produce a significant step forward in mosquito surveillance and control. In 2022, a novel system named VECTRACK (Irideon SL. Barcelona, Spain; www.irideon.es), based on a specific optical sensor mounted on a commercial BG-Mosquitaire trap (Biogents AG, Regensburg, Germany) and combined with a supervised ML algorithm, was developed for automatic counting and identification of *Aedes* and *Culex* mosquitoes by genus and sex [24]. The optical sensor includes an emitter of a collimated beam of near-infrared light and a receiver panel, which face each other through a transparent circular tube defined as the sensing zone. While entering the traps, mosquitoes pass through the sensing zone, casting a fast-changing shadow upon the optical receiver because of the modulation of the light beam by their wing flaps. This signal is automatically detected and recorded by the sensor, along with the GPS coordinates, date, exact time, ambient temperature and relative humidity at the instant of each capture. González-Pérez et al. extracted five features from each wingbeat recording via an application of digital signal processing method and analyzed these by different ML algorithms. After training and validation phases, the highest accuracy achieved during the testing phase performed on laboratory-reared mosquitoes was 94.2% for the identification of *Culex* vs. *Aedes* mosquitoes and > 99% for sex classification in both mosquito genera. When tested for the first time in the field to our knowledge—in two sites in Barcellona province (Spain) with predominance of *Cx. pipiens* in sympatry with *Ae. albopictus*—the system discriminated the target mosquito genera from other non-target insects with a balanced accuracy of 95.5% and classified the genus and sex of those mosquitoes with a balanced accuracy of 88.8% [25].

In this article, we report the results of the first extensive field evaluation of the VECTRACK system carried out in four regions across Italy with predominance of *Ae. albopictus* in sympatry with *Cx. pipiens* and compare the relative capture rates associated with different trap types. This allowed us to (i) evaluate whether the equipment of a commercial BG-trap for adult mosquitoes with VECTRACK sensor affected the catching capacity of the trap, (ii) estimate BG-trap (either equipped or not with VECTRACK) capture rate for *Ae. albopictus* based on known capture rate of sticky traps and (iii) assess the accuracy of the VECTRACK algorithm in correctly counting and classifying field *Aedes* and *Culex* mosquitoes by genus and sex.

## Methods

### Experimental design

The experiments were carried out in four urban and suburban areas across Italy in summer 2023 during the months of peak mosquito activity from July to August in Bergamo (north, Lombardy Region; GPS coordinates: 45°42′11″N, 9°49′58″E) and Rome (center, Lazio Region; 41°54′12″N, 12°30′51″E); from August to September in Padua (northeast, Veneto Region; 45°20′55″N, 11°57′14″E) and Procida Island (south, Campania Region, Naples; 40°45′38″N, 14°01′10″E).

**Fig. 1:**
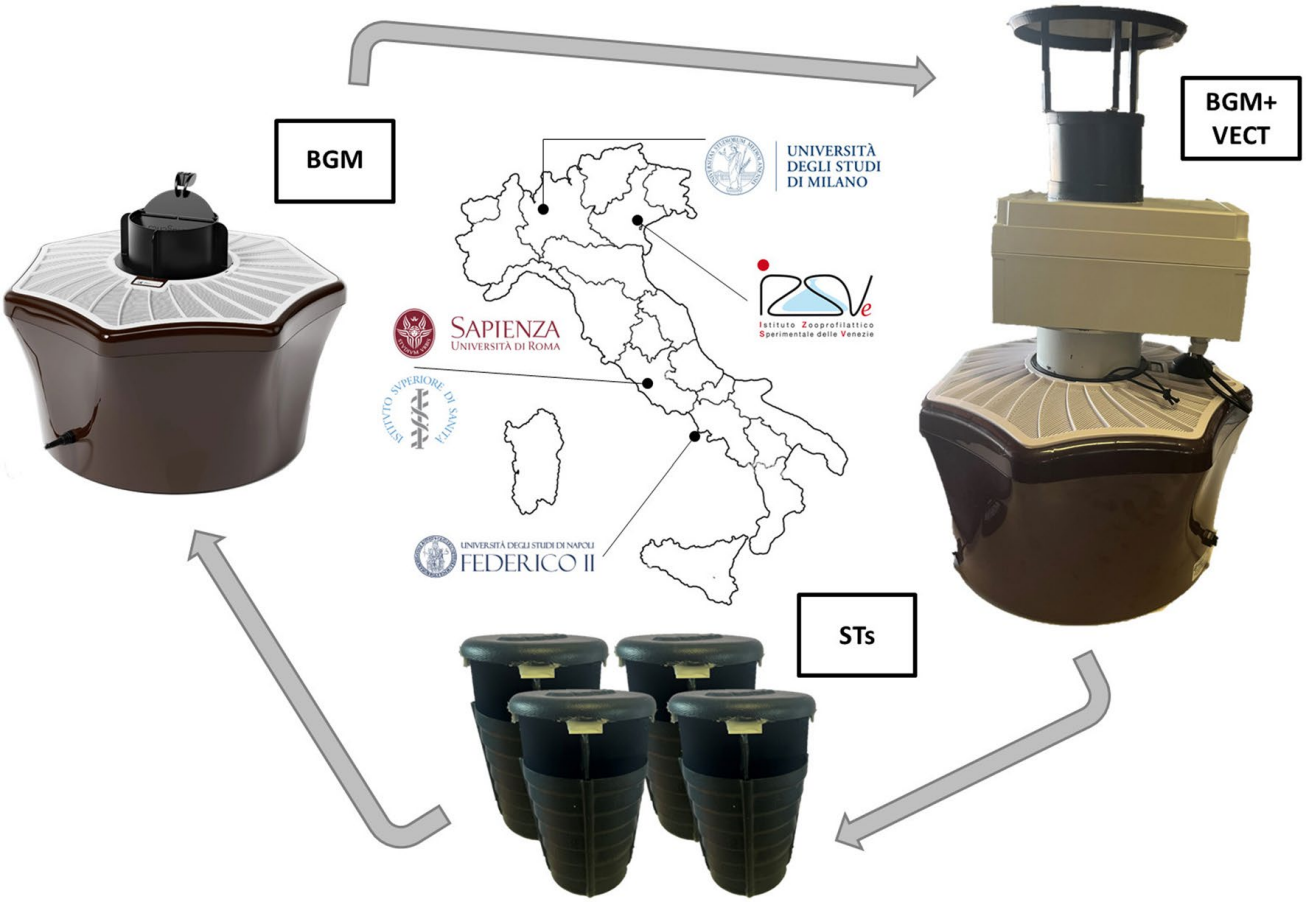
Experimental design of VECTRACK testing in Italy. Three types of traps were compared: BG-Mosquitaire (Biogents AG; BGM); BG-Mosquitaire equipped with the VECTRACK sensor (Irideon SL; BGM + VECT); four sticky traps (STs; [26]). Each trap was located in one site and moved to a close site (100–400 m) after 48 h following a predefined order of rotation. The complete trap rotation was performed in 6 days. Two additional replicates were conducted in the following weeks in each study area. The same experimental design was implemented in four regions (black dots in the map): Lombardy (by the University of Milan), Veneto (by the Istituto Zooprofilattico Sperimentale delle Venezie), Lazio (by the Sapienza University of Rome and Istituto Superiore di Sanità) and Campania (by the University of Naples Federico II)

Three sites were selected in each study area, and three types of traps were used and rotated each 48 h during the experiment (Fig. 1): (i) one BG-Mosquitaire (BGM), a commercial suction fan trap from Biogents AG (Regensburg, Germany) equipped with BG-Sweetscent chemical attractant (Biogents AG); (ii) one BGM equipped as the former plus the VECTRACK sensor, directly placed on the entrance of the trap (hereafter, BGM + VECT); (iii) four sticky traps (STs, [26]) located at 50–100 m from one another (the choice to deploy four STs instead of one was due to their limited capture performance, and they were counted as a single trapping event). Sticky traps were included in the experimental design to provide estimates of BGM and BGM + VECT capture rates based on the known capture rate for ST [22, 27]. The distance between sites (> 100 m and < 400 m) within each area was chosen to limit biases due to possible competition between traps while avoiding prolonged interruption of captures while rotating the traps among sites. The specific location of the traps at each site was selected to provide shade, nearby vegetation, shelter from rain and wind, access to electrical power and protection from theft. The complete trap rotation was performed within 6 days, and three replicates/area were conducted in the following weeks. Collected mosquitoes were counted and morphologically classified [28] by trained entomologists. Hereafter, we refer to this quantity as the number of “morphologically identified mosquitoes” to differentiate it from the number of mosquitoes counted and identified by VECTRACK ML algorithm.

### Statistical analyses

Analyses were carried out using R version 4.3.2, with the “glmmTMB” package [29].

#### Assessment of depletion effect

We checked potential depletion effects on the mosquito population caused by the continuous and repeated collections conducted in each site. To do this, we applied a generalized linear mixed model (GLMM) considering the total number of captured and morphologically identified mosquitoes as the response variable and the number of days from the first collection in the site, the trap type and their interaction as covariates. We considered as random effect the site nested within the region. The response variable was assumed to follow a negative binomial distribution, using a log link.

#### Assessment of changes in BG-Mosquitaire trapping performance due to VECTRACK application

We investigated whether equipping BGM with a VECTRACK sensor changed its trapping capability. To do this, we compared the total number of collected mosquitoes (as well as the total number of either *Ae. albopictus* or *Cx. pipiens*, i.e. the only two species currently identifiable by VECTRACK) morphologically identified in BGM and in BGM + VECT. At the species level, we also compared the estimated number of males and females separately. For each analysis, we applied a GLMM assuming a negative binomial distribution for the response variable with log link, the trap type as covariate and two crossed random effects: the sampling period and the site nested within region.

#### Assessment of VECTRACK performances in species and sex identification

We compared the number of morphologically identified mosquitoes collected by BGM + VECT with the number of mosquitoes identified by VECTRACK algorithm, considering the overall number of identified mosquitoes, the number of *Ae. albopictus* and *Cx. pipiens* specimens and the number of males and females separately identified for these two species. For *Ae. albopictus* and *Cx. pipiens* specimens and their sexes, we also provide the balance accuracy (BA) metric as defined by González-Pérez et al. (2024), where BA = Se + Sp/2, and Se and Sp indicate the sensitivity (the proportion of positive specimens correctly classified) and specificity (the proportion of negative specimens correctly classified), respectively. We computed the Spearman’s correlation coefficients corrected for ties, and we fitted a linear regression model considering the number of morphologically identified mosquitoes as the response variable and the number of mosquitoes identified by VECTRACK algorithm as the covariate.

## Results

**Fig. 2:**
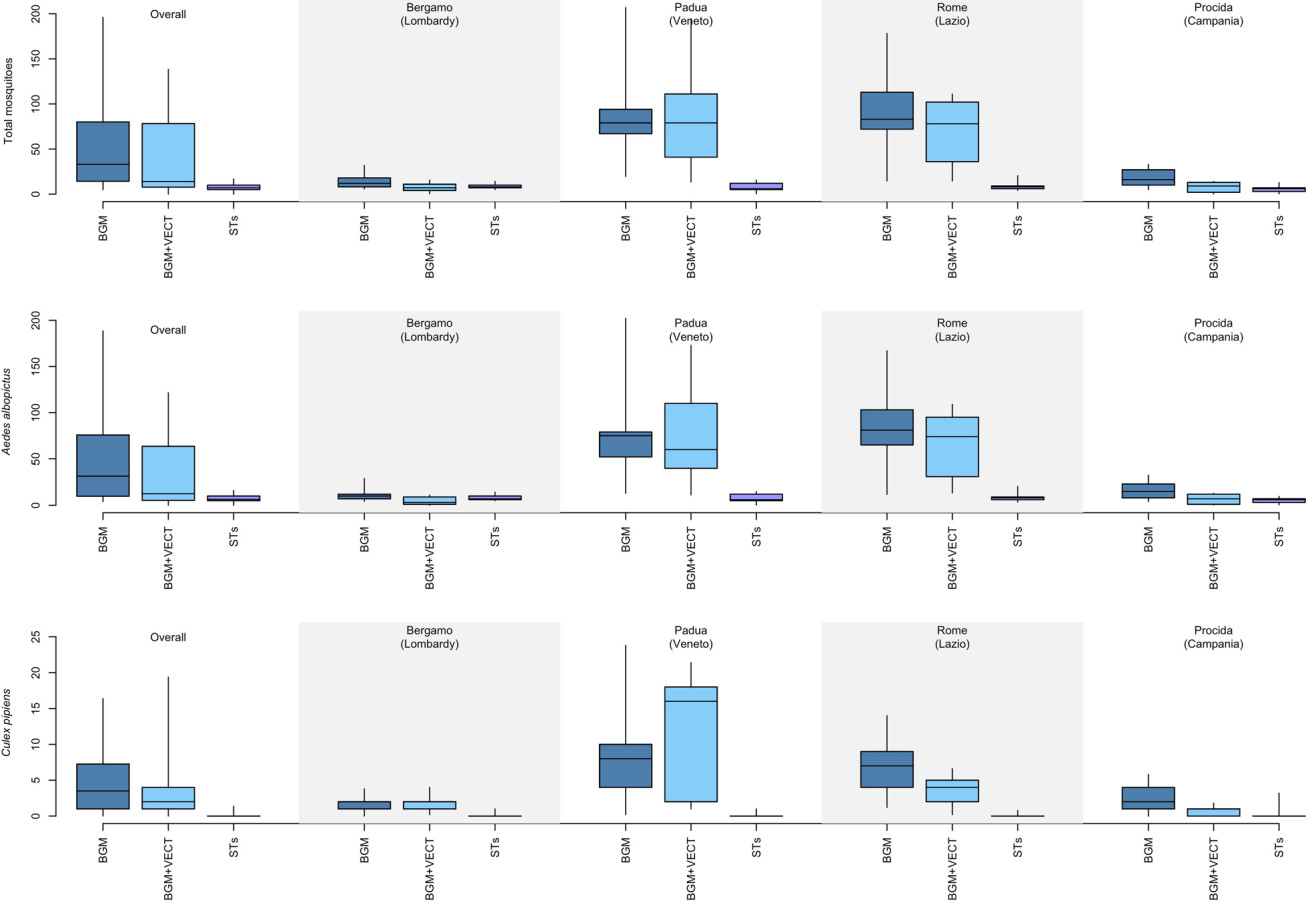
Box plots of total mosquito (upper panel), *Aedes albopictus* (central panel) and *Culex pipiens* (lower panel) captures for each type of collection overall and in each study area in Italy. Dark blue: BG-Mosquitaire (Biogents AG; BGM); light blue: BG-Mosquitaire equipped with the VECTRACK sensor (Irideon SL; BGM + VECT); purple: sticky traps ([26], STs; *N* = 4; considered as a single trap)

A total of 3829 mosquitoes were captured by all traps in the four field areas, corresponding to 18 collection-days/trap type/site (1998 by BGM, 1554 by BGM + VECT and 277 by STs). All collected mosquitoes were successfully morphologically identified by trained entomologists: *Aedes albopictus* was the predominant species (90.2%) followed by *Cx. pipiens* (8.8%); 36 other specimens were collected (23 individuals belonging to other *Aedes* species, 12 *Culiseta longiareolata* and one not-identified specimen; Table 1). Figure 2 shows box plots of *Ae. albopictus* and *Cx. pipiens* captures for each type of collection in each area.

**Table 1:** Total of Culicidae captured by three types of traps during 36 48-h-long trapping sessions in four areas across Italy and morphologically assigned to species and sex

Species	BGM + VECT			BGM			STs		
	*Total*	*Females*	*Males*	*Total*	*Females*	*males*	*Total*	*Females*	*Males*
*Aedes albopictus*	1389	1063	325	1800	1298	497	265	221	37
*Culex pipiens*	154	90	63	176	122	53	9	6	3
*Aedes koreicus*	2	1	1	10	9	1	1	0	1
*Aedes japonicus*	5	4	1	1	1	0	2	1	1
*Culiseta longiareolata*	2	0	2	10	0	10	0	0	0
*Aedes caspius*	1	–	–	0	–	–	0	–	–
*Aedes vexans*	0	–	–	1	–	–	0	–	–
*Other species*	1	0	0	0	0	0	0	0	0
Total	1554	1159	393	1998	1431	562	277	228	42

Three types of traps: BG-Mosquitaire (Biogents AG; BGM), BG-Mosquitaire equipped with the VECTRACK sensor (Irideon SL; BGM + VECT) and four sticky traps (STs; [26])

No relevant evidence of a depletion effect due to repeated captures at the sites was detected (Additional file 1: Fig. S1, Table S1).

### BGM trapping performance following VECTRACK sensor application and calculation of capture rate

According to GLMM results, the total number of mosquitoes collected by BGM + VECT was on average 32.3% lower (95% CI 2.1–53.1%, *P*: 0.038) than BGM (Table 2). Similar performances were estimated for the total number of *Ae. albopictus* adults captured (33.8%, 95% CI 1.9–55.4%; *P*: 0.040) and of *Ae. albopictus* males (35.1%, 95% CI 2.3–56.8%, *P*: 0.038). However, we did not find evidence against the hypothesis of comparable trapping performance between the BGM and BGM + VECT when considering *Ae. albopictus* females, *Cx. pipiens* adults, *Cx. pipiens* males and *Cx. pipiens* females (Table 2).

**Table 2:** Results of the generalized linear mixed models assessing difference in the number of captured mosquitoes between trap types

Response	Trap type	exp(beta)	95% confidence intervals	*P*-value
Total mosquitoes	BGM + VECT	0.677	0.469, 0.979	0.038
	ST	0.210	0.141, 0.314	< 0.001
Total *Aedes albopictus*	BGM + VECT	0.662	0.446, 0.981	0.040
	ST	0.233	0.152, 0.358	< 0.001
Total *Culex pipiens*	BGM + VECT	0.773	0.493, 1.212	0.3
	ST	0.054	0.024, 0.119	< 0.001
*Aedes albopictus* females	BGM + VECT	0.707	0.464, 1.077	0.11
	ST	0.276	0.174, 0.438	< 0.001
*Aedes albopictus* males	BGM + VECT	0.649	0.432, 0.977	0.038
	ST	0.083	0.048, 0.143	< 0.001
*Culex pipiens* females	BGM + VECT	0.625	0.376, 1.038	0.070
	ST	0.048	0.019, 0.123	< 0.001
*Culex pipiens* males	BGM + VECT	1.066	0.493, 2.308	0.9
	ST	0.059	0.015, 0.227	< 0.001

The BG-Mosquitaire (Biogents AG; BGM) trap is taken as reference, and its average value is not reported. The reported parameter values and their 95% confidence intervals are exponentiated and represent the multiplicative factor by which the expected number of captured mosquitoes changes depending on the trap type: BG-Mosquitaire equipped with the VECTRACK sensor (Irideon SL; BGM + VECT); sticky trap (ST; [26])

Results showed that the group of four STs/site consistently captured fewer mosquitoes than each BGM, irrespectively of the species or sex (Table 2). Interestingly, the BGM and BGM + VECT showed a multiplicative factor of 14.5 (95% confidence interval: 9.1–23.0) and 10.2 (95% confidence interval: 6.3–16.6) higher captures of *Ae. albopictus* females compared to a single ST. The capture rate of a single ST for *Ae. albopictus* (defined as the ratio between the number collected adult females and the number of adult females present within the flight range of this mosquito species) was previously estimated though mark-release-recapture experiments to be on average 1.838*10^−4^ [22, 27]. Consequently, according to our results, the capture rates of BGM and BGM + VECT are expected to range from 1.678*10^−3^ to 4.219*10^−3^ and from 1.161*10^−3^ to 3.045*10^−3^, respectively.

### VECTRACK algorithm performance in *Aedes albopictus* and *Culex pipiens* identification

To assess VECTRACK algorithm performance in the identification of mosquito species, we focused only on the collections made by the BGM + VECT and compared the number of *Ae. albopictus* and *Cx. pipiens* morphologically identified by an expert operator with the identification provided by VECTRACK algorithm. Notably, the number of specimens belonging to other Culicidae species (not targeted by VECTRACK algorithm) was negligible (*N* = 11).

Overall, VECTRACK algorithm counted 53 fewer *Ae. albopictus* and *Cx. pipiens* in the trap compared to an actual number of 1543 morphologically identified specimens (Table 3). This difference amounts to 3.4% unidentified *Ae. albopictus* and *Cx. pipiens* regarding the total captures. VECTRACK algorithm counted 5% (*N* = 69) *Ae. albopictus* less than the operator and overestimated the number of *Cx. pipiens* by 10.4% (*N* = 16). This amounts to a balance accuracy (as defined in González-Pérez et al.) of 99.8% and 99.4%, respectively.

**Table 3:** Mosquitoes identified by operator-mediated morphological examination (M) or by VECTRACK algorithm (VT)

Species	Total			Females			Males		
	M	VT	Difference (%)	M	VT	Difference (%)	M	VT	Difference (%)
*Aedes albopictus*	1389	1320	-69 (-5%)	1063	820	-243 (-22.9%)	325	500	175 (53.8%)
*Culex pipiens*	154	170	16 (10.4%)	90	111	21 (23.3%)	63	59	-4 (-6.4%)
Total	1543	1490	-53 (-3.4%)	1153	931	222 (-19.3%)	388	559	171 (44.1%)

**Fig. 3:**
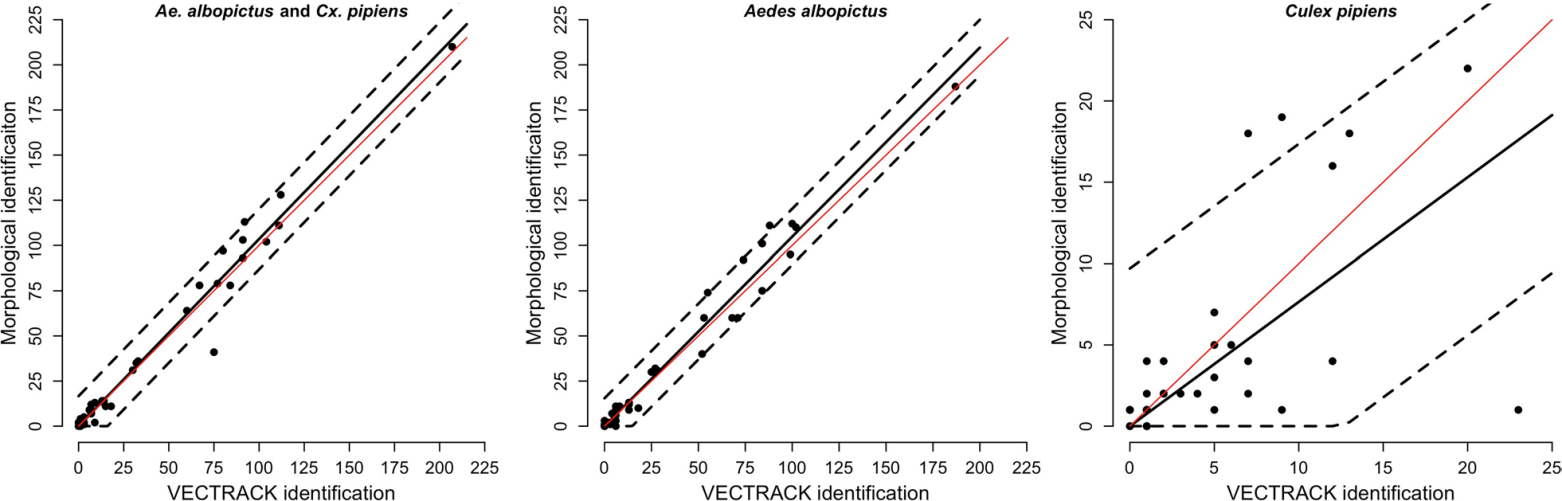
Relationship between the number of captured target species, *Aedes albopictus* or *Culex pipiens*, as identified by VECTRACK algorithm and by operator-mediated morphological examination. Points correspond to the total number of collected adult mosquitoes (left panel), total number of collected *Ae. albopictus* adults (central panel) and total number of collected *Cx. pipiens* adults (right panel) by the sensor (x axis) and operator (y axis). The black line represents the mean predicted values obtained with the considered linear regression; the dashed lines represent the corresponding 95% prediction interval. The red diagonal line represents the ideal perfect identification performance

Overall, results show a very high correlation between the two identification methods applied across different sites and at different times for the total number of *Ae. albopictus* and *Cx. pipiens* (Spearman’s correlation coefficient, rs = 0.96, *P* < 0.0001). Results from the regression model (Table S2) estimated a 1.03 (95% CI 0.99–1.08) relationship between the overall number of adult mosquitoes identified by VECTRACK algorithm and by the expert entomologist, meaning that for every 100 *Ae. albopictus* and *Cx. pipiens* identified by VECTRACK algorithm, the expectation is to have captured on average 103 (SD: 8.5) *Ae. albopictus* and *Cx. pipiens* (Fig. 3).

Similar good performance was obtained for *Ae. albopictus* with a correlation of 0.96 (*P* < 0.0001) between the two identification methods. Overall, the number of morphologically identified *Ae. albopictus* was comparable to the number provided by VECTRACK algorithm (Chi-square test, *χ*^2^ = 0.409, *df* = 1, *P* = 0.52). Results from the linear model estimated a 1.05 (95% CI 1–1.1) relationship between the number of *Ae. albopictus* identified by VECTRACK and by the operator corresponding to an expectation of 105 (SD: 7.9) *Ae. albopictus* for every 100 *Ae. albopictus* identified by VECTRACK algorithm (Fig. 3).

The number of identified *Cx. pipiens* was comparable between operator and VECTRACK algorithm (Chi-square test, *χ*^2^ = 1.64, *df* = 1, *P* = 0.2). However, a less strong correlation was present (0.70, *P* < 0.0001) with VECTRACK algorithm overestimating the number of *Cx. pipiens* and showing high residual uncertainty regarding the number of captured specimens. For every 100 *Cx. pipiens* identified by VECTRACK algorithm, we expect to have captured on average 77 (SD: 5) *Cx. pipiens* (Fig. 3).

### VECTRACK algorithm performance in *Aedes albopictus* and *Culex pipiens* female and male identification

Overall, VECTRACK algorithm provides high balance accuracy for females (99.1% and 98.8% for *Ae. albopictus* and *Cx. pipiens*, respectively), but a lower value for males was observed (62.6% and 62.8%). In particular, VECTRACK algorithm underestimated the number of *Ae. albopictus* females (– 22.9%) and overestimated the number of males (53.8%), while an opposite trend was observed for *Cx. pipiens* mosquitoes (23.3% overestimation of females and – 6.4% underestimation of males).

For *Ae. albopictus*, a very high correlation between estimates provided by operators and VECTRACK algorithm was found for both females (Spearman’s correlation coefficient, *rs *= 0.97; *P* < 0.0001) and males (rs = 0.89 *P* < 0.0001). However, a significant difference was found in the identification of sexes between the two identification methods (Chi-square test, *χ*^2^ = 66.1, *df* = 1, *P* < 0.001). The misidentification of *Ae. albopictus* females and overestimation of *Ae. albopictus* males was found to be systematic and consistent across sites.

**Fig. 4:**
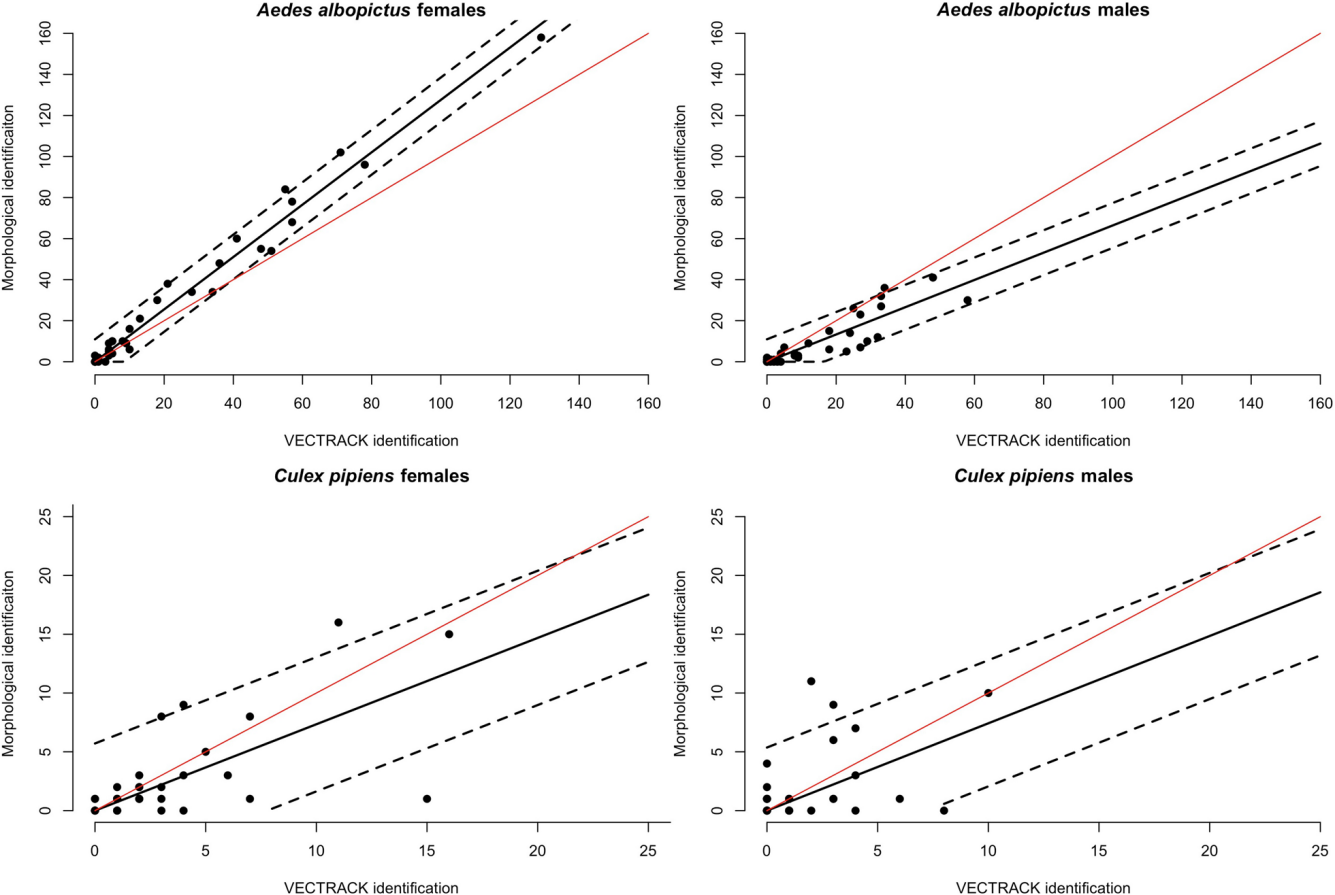
Observed numbers (dots) of *Aedes albopictus* (left panel) and *Culex pipiens* females (right panel) counted by VECTRACK algorithm (x axis) and by operator-mediated morphological identification (y axis). The black line represents the mean predicted values by the linear regression and the dashed line the 95% prediction interval. The diagonal red line represents the ideal perfect identification performance

The estimated relationship between *Ae. albopictus* females counts by VECTRACK algorithm and by operator-mediated morphological identification is 1.28 (95% CI 1.23–1.33) and was found to correct the estimate of VECTRACK algorithm providing an estimate of 128 (SD: 5.6) actual catches for every 100 females identified by VECTRACK algorithm (Fig. 4). The estimated relationship between *Ae. albopictus* male counts by VECTRACK algorithm and the operator is lower 0.66 (95% CI: 0.57–0.76).

We did not find evidence against the hypothesis of a similar identification performance of *Cx. pipiens* sexes between VECTRACK algorithm and by operator-mediated morphological examination (Chi-square test, *χ*^2^ = 1.17, *df* = 1, *P* = 0.28). However, the correlation between estimates provided by the two identification methods was found to be lower for both females (Spearman’s correlation coefficient, rs = 0.67 *P* < 0.0001) and males (rs = 0.37, *P* = 0.025).

## Discussion

Previous studies showed the performance of the VECTRACK system in identifying *Ae. albopictus* and *Cx. pipiens* mosquitoes reared under laboratory conditions [24] as well as in field experiments carried out in two sites in Spain with prevalence of *Cx. pipiens* [25]. We extended the testing of the VECTRACK system to four experimental areas from northern to southern Italy where *Ae. albopictus* prevails and implemented an experimental design including rotation of BGM, BGM + VECT and sticky traps in each site. This allowed us to assess not only the performance of the VECTRACK algorithm in identifying the target mosquitoes under different eco-climatic conditions but also the capture capacity of the system compared to conventional BGM traps widely adopted for mosquito monitoring. We also included ST collections in the experimental design, which are known to yield fewer *Ae. albopictus* female catches than traps targeting host-seeking females but have relevant operation advantages (e.g. lower costs, no need of power supplies and protection from rain and thefts), to provide estimates of BGM and BGM + VECT capture rates based on the known capture rate for ST [22, 27].

The first question was whether the equipment of the BGM trap with the VECTRACK sensor could affect the capture capacity of the trap, possibly because of a lower aspiration strength or a higher point of entrance. Although the total number of mosquitoes collected by BGM + VECT was on average lower compared to BGM, the results obtained by GLMM did not highlight a significant reduction in the collections of *Ae. albopictus* females and *Cx. pipiens* females and males. Only *Ae. albopictus* male captures were significantly lower in BGM + VECT. Notably, the BGM + VECT system has a huge advantage compared to conventional adult traps by offering the possibility to be used continuously over time with very low maintenance, as operators receive the capture data directly on their PCs. Thus, even if the BGM + VECT system might collect significantly fewer mosquitoes than BG traps, this would not reduce its very high potential for field study and monitoring. However, results demonstrate the very good performance of BGM + VECT in mosquito collection and also suggest that the sensor could moderately affect the BG performance as the collected mosquito has to pass into a 20 cm-long tube before being trapped. This evidence could lead to further improvement of the BGM + VECT system by slightly increasing the aspiration strength. The estimates we provided for the capture rates associated with different traps would allow appropriate adjustments for estimating the mosquito abundance in sites of data collection.

Second, the results obtained allowed us to quantify the heterogeneous effectiveness associated with different trap types in collecting female mosquitoes and predict estimates of absolute mosquito density. The obtained estimates agree with the relative capture rate estimated for ST and BG traps recently obtained by simultaneously fitting mosquito data collected with different trap types [22]. Mark-release-recapture experiments conducted in Italy [27] suggested that collecting 10 *Ae. albopictus* adult females with a ST over a 24-h period corresponds to a local density of 4332 (95% CI 3272–5884) females per hectare. Based on this result, the estimates from the present experiment suggest that collecting 10 *Ae. albopictus* females in a BGM over a 24-h period may correspond to a local density of 299 (95% CI 189–475) of adult females per hectare, while 10 *Ae. albopictus* females collected in a BGM + VECT may correspond to a density of 423 (95% CI 261–686) adult females per hectare.

Third, the capacity of the VECTRACK algorithm to identify target mosquito species was tested. It should be highlighted here that the VECTRACK machine learning algorithm has been trained to identify *Ae. albopictus, Aedes aegypti* and *Cx. pipiens* so far [24] and that previous studies have reported the results of the identification performance at the genus level [24, 25]. Here, we report the results at the species level, as *Ae. aegypti* is not present in Italy and *Cx. pipiens* was the only species of genus *Culex* found in the collections. In other words, we did not include the 11 mosquito specimens belonging to other species in the analyses, but due to the small sampling size, the effect of this is negligible. Overall, the VECTRACK species identification showed > 99% balance accuracy, with a 5% underestimation of *Ae. albopictus* and 10.4% overestimation of *Cx. pipiens*. These values are consistent with results from Spain, showing a balance accuracy of ~ 95%, and 7.6% lower counts by VECTRACK algorithm compared to morphological identifications [25]. The correlation between the two identification methods applied across different sites was very high for *Ae. albopictus* (0.96) and lower for *Cx. pipiens* (0.70).

Moreover, the capacity of the VECTRACK algorithm to identify the sex of the target mosquito species was tested. Results show very high balance accuracy for females of both species (~ 99%), a value higher than those (87%–93%) calculated in the Spanish field experiments. However, balance accuracy for males (~ 62%) is lower than in previous trials (80–95%). The correlation between sex identification by operators and VECTRACK was high for both *Ae. albopictus* females and males (Spearman’s correlation coefficient, rs > 0.89), although a significant systematic misidentification of sexes was found in all sampling areas. However, the correlation was lower for *Cx. pipiens* females (Spearman’s correlation coefficient, *rs* = 0.67) and males (*rs* = 0.37).

Finally, the output of the linear model allows to predict the ranges of females and males of both species actually collected, based on the number of mosquitoes identified by VECTRACK algorithm. For instance, the model estimates that (with a 95% prediction interval) for every 100 *Ae. albopictus* or *Cx. pipiens* identified by VECTRACK algorithm, the actual catches are 89–120 or 67–86. These estimates are in the range of the variability observed by simply positioning the trap in a different nearby location or on a different day of the week.

Overall, the obtained results strongly support the VECTRACK system as a powerful tool for mosquito monitoring and study and its applicability over a range of ecological conditions. Moreover, Irideon company is further training the machine learning VECTRACK algorithm and has already released an updated version, which we preliminarily tested, showing improved sex identification for *Ae. albopictus*, with underestimation of the number of females reduced from – 22.9% to – 11.8%, and overestimation of the number of males reduced from 53.8% to 17.2% (data not shown). It is also important to highlight that the system is designed to work 24 h/day, 7 days/week with minimum maintenance and that this can lead to a significant increase of collected data while simultaneously reducing human effort and the associated costs. Indeed, at present, its use should be restricted to areas with limited presence of species different from *Ae. albopictus* and *Cx. pipiens*, such as most urban areas in Mediterranean Europe. At this stage, it is not possible to predict whether the algorithm performance would be reduced at high frequencies of other mosquito species which it has never been trained for. However, the VECTRACK ML algorithm is being trained to identify other species, which will eventually make its exploitation possible in different geographical regions or as a sentinel trap in potential points of entry.

In addition, the VECTRACK system has other invaluable features, as it collects data on mosquito capture, as well as temperature and humidity, in real time. This allows us to study features of mosquito bionomics virtually impossible to be studied with conventional monitoring tools, such as the daily activity patterns of the target mosquitoes, whose knowledge would be instrumental for optimized mosquito control interventions. The network of research groups collaborating within the IN-FACT project (Extended Partnership Initiative on Emerging Infectious Diseases; MUR project no. PE00000007) is taking advantage of this unprecedented opportunity to implement a large-scale study based on the VECTRACK system on *Ae. albopictus* and *Cx. pipiens* circadian rhythm patterns across different eco-climatic conditions from northern to southern Italy in 2024–2025.

## Supplementary Information

Additional file 1: Fig. S1 Potential depletion of mosquitoes collected in each trap type after repeated sampling in each location. Each trap was analyzed separately because of the potentially different capture rate. Points are observed values; dashed line is a simple linear regression fit to help visualize a potential trend.

Additional file 2: Table S1 Results of the generalized linear mixed models assessing potential depletion after repeated mosquito collections. BG-Mosquitaire trap (BGM) considered as reference. Parameters values and their 95% confidence intervals are not exponentiated.

Additional file 3: Table S2 Estimated linear relationship between operator-based morphological identification and identification by VECTRACK algorithm of females and males of *Aedes albopictus *and* Culex pipiens*.

## Data Availability

The dataset used and analyzed in the study is available from the corresponding author on reasonable request.

## Abbreviations

DENV: Dengue virus
CHIKV: Chikungunya virus
WNV: West Nile virus
MBDs: Mosquito-borne diseases
BGM: BG-Mosquitaire trap
BGM + VECT: BG-Mosquitaire trap equipped with VECTRACK sensor
ST: Sticky trap
