# Supplementary figures and images for: An easier life to come for mosquito researchers: field-testing across Italy supports VECTRACK system for automatic counting, identification and absolute density estimation of Aedes albopictus and Culex pipiens adults
Source: Parasit Vectors. 2024 Oct 2;17:409. doi: 10.1186/s13071-024-06479-z (PMC11448096; doi:10.1186/s13071-024-06479-z)

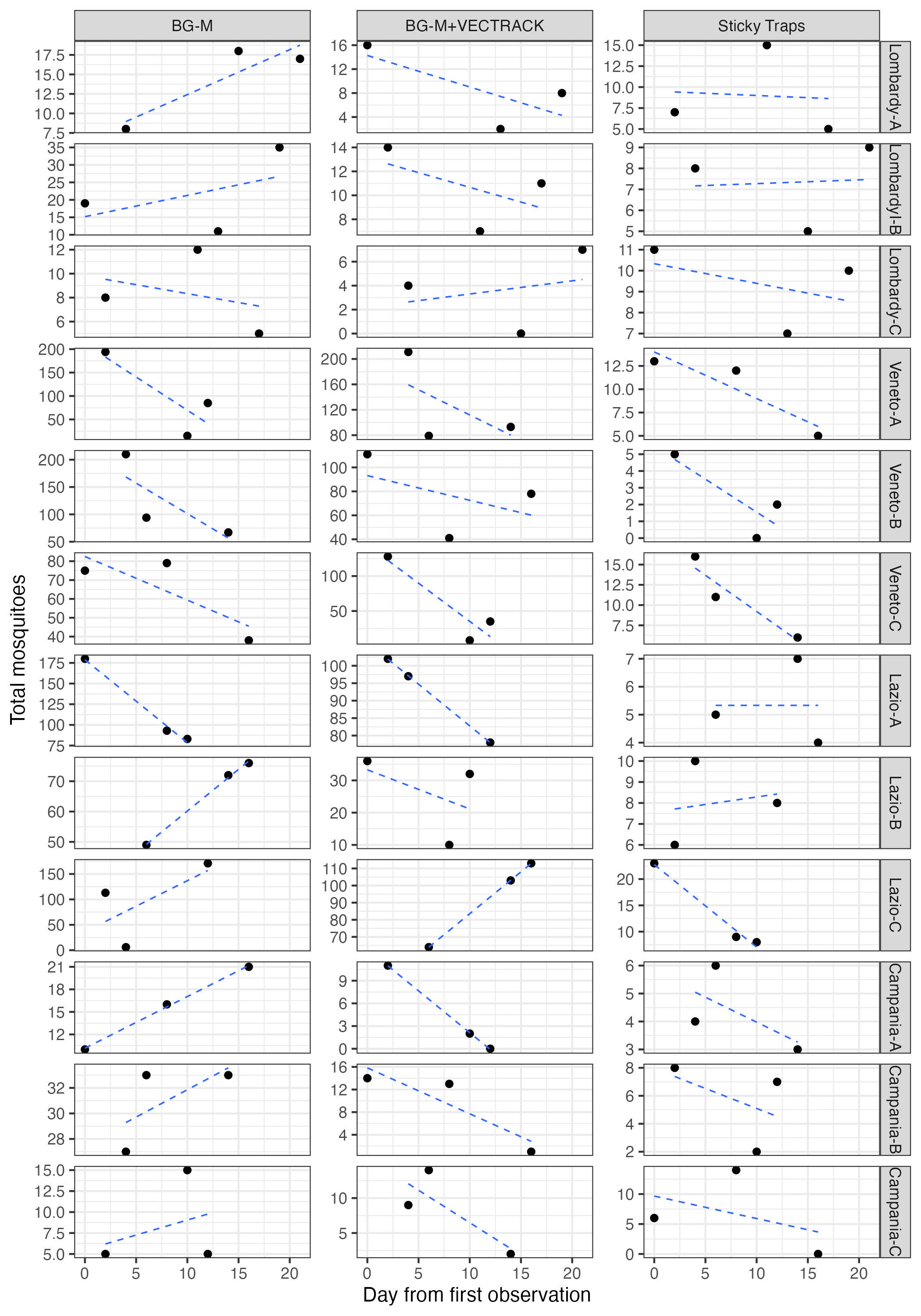

Supplement: Supplementary file 1 — Additional file 1: Fig. S1 Potential depletion of mosquitoes collected in each trap type after repeated sampling in each location. Each trap was analyzed separately because of the potentially different capture rate. Points are observed values; dashed line is a simple linear regression fit to help visualize a potential trend. [file 13071_2024_6479_MOESM1_ESM.jpeg]
