# Supplementary material for: An easier life to come for mosquito researchers: field-testing across Italy supports VECTRACK system for automatic counting, identification and absolute density estimation of Aedes albopictus and Culex pipiens adults
Source: Parasit Vectors. 2024 Oct 2;17:409. doi: 10.1186/s13071-024-06479-z (PMC11448096; doi:10.1186/s13071-024-06479-z)
Supplement: Supplementary file 2 — Additional file 2: Table S1 Results of the generalized linear mixed models assessing potential depletion after repeated mosquito collections. BG-Mosquitaire trap (BGM) considered as reference. Parameters values and their 95% confidence intervals are not exponentiated. [file 13071_2024_6479_MOESM2_ESM.docx]

**An easier life to come for mosquito researchers: field-testing across Italy supports VECTRACK system for automatic counting, identification and absolute density estimation of *Aedes albopictus* and *Culex* *pipiens* adults.**

**Martina Micocci^1†^, Mattia Manica^2†^, Ilaria Bernardini^3^, Laura Soresinetti^4^, Marianna Varone^5^, Paola Di Lillo^5^, Beniamino Caputo^1^, Piero Poletti^2^, Francesco Severini^3^, Fabrizio Montarsi^6^, Sara Epis^4^, Marco Salvemini^5^, Alessandra della Torre^1*^**

^†^Martina Micocci and Mattia Manica contributed equally to this work.

_1_ Sapienza University of Rome, Department of Public Health and Infectious Diseases, Rome, Italy

_2_ Fondazione Bruno Kessler, Center for Health Emergencies, Trento, Italy

_3_ Istituto Superiore di Sanità, Department of Infectious Diseases, Rome, Italy

_4_ University of Milan, Department of Biosciences and Pediatric Clinical Research Center “Romeo ed Enrica Invernizzi”, Milan, Italy

_5_ University of Naples Federico II, Department of Biology, Naples, Italy

_6_ Istituto Zooprofilattico Sperimentale delle Venezie, Legnaro, Italy

^*^Correspondence: alessandra.dellatorre@uniroma1.it

MMi: martina.micocci@uniroma1.it

MMa:

mmanica@fbk.eu
IB: ilaria.bernardini@iss.it
LS: laura.soresinetti@unimi.it

MV: marianna.varone@unina.it
PDL: paola.dilillo01@universitadipavia.it

BC: beniamino.caputo@uniroma1.it
PP: poletti@fbk.eu

FS: francesco.severini@iss.it
FM: [fmontarsi@izsvenezie.it](mailto:fmontarsi@izsvenezie.it)

SE: sara.epis@unimi.it

MS: marco.salvemini@unina.it

**Supplementary information**

**Additional file 1: Fig. S1 Potential depletion of mosquitoes collected in each trap type after repeated sampling in each location.** Each trap was analysed separately due to the potentially different capture rate. Points are observed values, dashed line is a simple linear regression fit to help visualize a potential trend.

**Additional file 2: Table S1 Results of the generalized linear mixed models assessing potential depletion after repeated mosquito collections**. BG-Mosquitaire trap (BGM) considered as reference. Parameters values and their 95% confidence intervals are not exponentiated.

| **Covariate** | **Parameters** | **95% confidence interval** | ***P*-value** |
| --- | --- | --- | --- |
| BGM | — | — | — |
| Day | -0.015 | -0.058, 0.029 | 0.504 |
| BGM-VECT | -0.158 | -0.787, 0.472 | 0.623 |
| Sticky | -1.572 | -2.242, -0.901 | <0.001 |
| Day * BGM-VECT | -0.030 | -0.091, 0.031 | 0.336 |
| Day * Sticky Trap | -0.001 | -0.063, 0.062 | 0.986 |
|  | | | |
