# Supplementary material for: An easier life to come for mosquito researchers: field-testing across Italy supports VECTRACK system for automatic counting, identification and absolute density estimation of Aedes albopictus and Culex pipiens adults
Source: Parasit Vectors. 2024 Oct 2;17:409. doi: 10.1186/s13071-024-06479-z (PMC11448096; doi:10.1186/s13071-024-06479-z)
Supplement: Supplementary file 3 — Additional file 3: Table S2 Estimated linear relationship between operator-based morphological identification and identification by VECTRACK algorithm of females and males of Aedes albopictus and Culex pipiens. [file 13071_2024_6479_MOESM3_ESM.docx]

**Additional file 3: Table S2 Estimated linear relationship between operator-based morphological identification and identification by** **VECTRACK algorithm** **of females and males of *Aedes albopictus* and *Culex pipiens*.**

| **Response** | **Parameter** | **Standard Error** | **T value** | ***P*-value** | **Standard Error** |
| --- | --- | --- | --- | --- | --- |
| Total mosquitoes | 1.035 | 0.0226 | 45.83 | <0.0001 | 8.487 |
| *Aedes albopictus* | 1.048 | 0.0233 | 44.89 | <0.0001 | 7.887 |
| *Culex pipiens* | 0.765 | 0.1141 | 6.703 | <0.0001 | 4.952 |
| *Aedes albopictus* females | 1.276 | 0.0252 | 50.54 | <0.0001 | 5.567 |
| *Aedes albopictus* males | 0.664 | 0.0453 | 14.08 | <0.0001 | 5.581 |
| *Culex pipiens* females | 0.734 | 0.0984 | 7.46 | <0.0001 | 2.914 |
| *Culex pipiens* males | 0.743 | 0.1583 | 4.69 | <0.0001 | 2.737 |
